# Application of photocrosslinkable hydrogels based on photolithography 3D bioprinting technology in bone tissue engineering

**DOI:** 10.1093/rb/rbad037

**Published:** 2023-04-24

**Authors:** Jianpeng Gao, Xiao Liu, Junyao Cheng, Junhao Deng, Zhenchuan Han, Ming Li, Xiumei Wang, Jianheng Liu, Licheng Zhang

**Affiliations:** Department of Orthopaedics, Chinese PLA General Hospital, Beijing 100036, China; Chinese PLA Medical School, Beijing 100036, China; Department of Orthopaedics, Chinese PLA General Hospital, Beijing 100036, China; Chinese PLA Medical School, Beijing 100036, China; Department of Orthopaedics, Chinese PLA General Hospital, Beijing 100036, China; Chinese PLA Medical School, Beijing 100036, China; Department of Orthopaedics, Chinese PLA General Hospital, Beijing 100036, China; Department of Orthopaedics, Chinese PLA General Hospital, Beijing 100036, China; Department of Orthopaedics, Chinese PLA General Hospital, Beijing 100036, China; State Key Laboratory of New Ceramics and Fine Processing, Key Laboratory of Advanced Materials of Ministry of Education, School of Materials Science and Engineering, Tsinghua University, Beijing 100084, China; Department of Orthopaedics, Chinese PLA General Hospital, Beijing 100036, China; Department of Orthopaedics, Chinese PLA General Hospital, Beijing 100036, China

**Keywords:** photocrosslinkable hydrogels, photolithography 3D bioprinting, bone tissue engineering, bone regeneration, bone defect

## Abstract

Bone tissue engineering (BTE) has been proven to be an effective method for the treatment of bone defects caused by different musculoskeletal disorders. Photocrosslinkable hydrogels (PCHs) with good biocompatibility and biodegradability can significantly promote the migration, proliferation and differentiation of cells and have been widely used in BTE. Moreover, photolithography 3D bioprinting technology can notably help PCHs-based scaffolds possess a biomimetic structure of natural bone, meeting the structural requirements of bone regeneration. Nanomaterials, cells, drugs and cytokines added into bioinks can enable different functionalization strategies for scaffolds to achieve the desired properties required for BTE. In this review, we demonstrate a brief introduction of the advantages of PCHs and photolithography-based 3D bioprinting technology and summarize their applications in BTE. Finally, the challenges and potential future approaches for bone defects are outlined.

## Graphical Abstract

**Figure:**
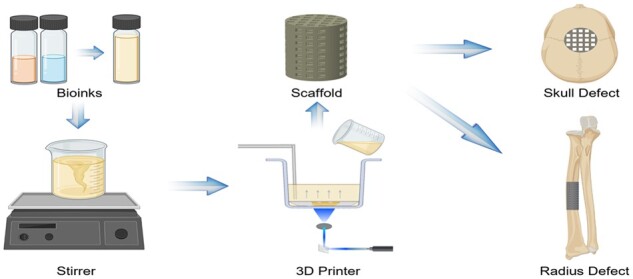

## Introduction

Bone defects caused by aging, disease, trauma and other factors do great harm to the human body [1]. Therefore, effective treatment is needed to achieve repair and regeneration. Natural bone is a complex natural biomineralization system consisting of water, organic and inorganic components. Among them, more than 90% of the organic components are type I collagen, and almost all the inorganic components are calcium phosphate [2]. Thus, bone grafting has been the gold standard for the treatment of bone defects in recent decades owing to the complexity of natural bone ingredients until the rapid development of bone tissue engineering (BTE), which is expected to be one of the alternative materials for autologous bone [3–6].

Hydrogels, as the main materials for BTE due to their good biocompatibility, are mainly divided into natural polymers (e.g. gelatin, hyaluronic acid, silk fibroin (SF), collagen, chitosan) with good biological functions and synthetic polymers (e.g. polyethylene glycol (PEG), polypropylene fumarate (PPF), F127, polycaprolactone (PCL), polylactic acid (PLA)) with good mechanical properties [7–9]. Moreover, the photocrosslinkable hydrogels (PCHs) formed after incorporating photoreactive moieties (e.g. methacrylate, acrylate) into hydrogels can realize the transformation from the liquid to the solid phase with photo-initiators and light exposure. Photopolymerization occurs when light-sensitive compounds interact with photo-initiators and light exposure to produce free radicals that initiate the process of polymerization to prepare a covalently crosslinked hydrogel [10]. Because of the complex composition of natural bone, PCHs are more commonly used in the combination of natural polymers and synthetic polymers to maintain biocompatibility while taking the degradation and mechanical properties into account [11].

Natural bone not only has complex ingredients but also possesses a highly special structure with different pore sizes and stepped porosity from inside to outside, which gives it better mechanical properties. Appropriate pore sizes can help cells better adhere, proliferate and differentiate, which is conducive to the formation of a functional vascular system and the transportation of bioactive factors [12, 13]. Conventional manufacturing technologies for BTE include solvent pouring/particle leaching, gas foaming, freeze drying, phase separation, electrospinning, three-dimensional (3D) printing, etc. [14–19]. Among them, 3D printing technology with computer-aided design (CAD) modeling has the highest accuracy and repeatability, as well as high spatiotemporal control of structures [20]. It is one of the most ideal preparation methods for clinical application. As one of the significant 3D printing methods, photolithography-based 3D bioprinting, such as digital light processing (DLP) and stereolithography (SLA), can be used to prepare BTE scaffolds with satisfactory mechanical properties and biocompatibility as well as bionic bone structures by bioinks laded with cells and bioactive factors to increase the specific biological function of BTE scaffolds [21–23]. Therefore, PCHs based on photolithography 3D bioprinting technology may provide a method for the treatment of complex bone defects in the clinic.

In this review, we demonstrate a brief introduction of the advantages of PCHs and photolithography-based 3D bioprinting technology, and summarize the applications in BTE. Finally, the challenges and potential future approaches for bone defects are outlined.

## Advantages of photocrosslinkable hydrogels in bone tissue engineering

Bone biomimetic scaffolds based on BTE make it possible to produce the next generation of biological implants capable of treating severe bone defects. 3D bioprinting enables excellent control of the geometry and macrostructure of bone biomimetic scaffolds, facilitating the construction of highly complex anisotropic tissue structures similar to those of natural bone, thereby mimicking its excellent mechanical properties [24, 25]. Bioinks have held the key to developing 3D structures for bone and cartilage defect repair. Due to their rapid *in situ* gelation, excellent biocompatibility and biodegradability, PCHs have shown many unique advantages as bioinks for biomimetic scaffold manufacturing [26, 27]. Derived from natural or synthetic polymers, PCHs can ensure biocompatibility in the presence of cells with minimal immunogenicity, thereby avoiding implant failure caused by immune or inflammatory responses [28]. The biodegradation properties enable proper tissue remodeling without harmful byproducts, allowing for *in situ* growth as well as the functional release of cells and cytokines, and possessing degradability matched to the rate of bone tissue regeneration [29]. Another major advantage of PCHs is the immediate response to light, thus inducing a rapid transition of structure or morphology. This *in situ* rapid gelation capability not only ensures the structural stability of the printed scaffolds but also enables the formation of complex biomimetic structures through precise spatiotemporal control [20]. PCHs contain different functional groups, which can enable different functionalization strategies for photocrosslinking to achieve the desired properties required for BTE. In addition, PCHs can be prepared by simple synthesis methods to effectively control the production cost, which is of great significance for product transformation and clinical applications.

In addition to structural bionics, functional biomimicry details should also be carefully considered when developing bone biomimetic materials. The key to the repair of bone lies in the migration and regeneration of osteocytes as well as the remodeling of the extracellular matrix [30]. Maintaining a balanced environment with cell viability, cytokine activity and mechanical integrity is critical for the construction of bone repair scaffolds. Precise control of crosslink density and physicochemical properties by adjusting light intensity and exposure time enables precise spatiotemporal control over the placement of cells and biomaterials. Thus, the PCHs can correctly simulate the 3D extracellular matrix environment to provide a nutrient environment suitable for cell proliferation and differentiation [31]. PCHs also provide a feasible solution for the precise and intelligent development of BTE. Photopolymerized hydrogels can form 3D patterns containing different bioactive components. Through modification or encapsulation with biofunctional moieties, a specific release of cytokines can be achieved to manipulate cellular behavior [32]. When implanted into the defect area, the hydrogels can be degraded by hydrolysis or enzymatic methods under preset spatiotemporal conditions to realize the directional delivery of cells, cytokines, drugs and other effectors [33]. All of these properties make PCHs uniquely advantageous for 3D printing of live cells and/or growth factors. Overall, PCHs, as highly promising 3D printing bioinks, will help to promote and broaden the utility of bone tissue-engineered repair materials to meet structural and functional bionic needs. Its manufacturing potential could help drive continued progress in the development of physiologically relevant biomimetic BTE.

## Photolithography-based 3D bioprinting for PCHs

3D bioprinting has emerged as a promising fabrication strategy for BTE, with effective control over the geometry and microstructures of scaffolds [34]. Using this technology can homogeneously encapsulate cells and growth factors into 3D scaffolds, effectively addressing the challenge of uneven cell distribution and limited cell density caused by seeding on traditional scaffolds [35, 36]. PCHs, responding to light and causing structural or morphological transformations, have been one of the gold standard materials for 3D bioprinting [34]. They are characterized by desirable elastic and hydrating properties, as well as an ECM-mimetic crosslinked network structure, allowing cells to survive and maintain their function [37].

To date, there are various 3D printing technologies based on light-curing [38], such as SLA, DLP, liquid crystal display and continuous liquid interface production, among which SLA and DLP are the leading lithography-based 3D bioprinting technologies in BTE [21]. Although neither of them can fully replicate the complexity of bone tissue, these two representative bioprinting technologies are widely used in the preparation of PCHs due to their good biocompatibility and facilitated combination of multiple crosslinking mechanisms [39, 40] (Table 1). Indeed, PCHs act as supportive and regulatory platforms for the cells entrapped within their networks during 3D bioprinting. This smart hydrogel and 3D technology provide a solid integration for the printing of multidimensional structures [41].

**Table 1.** The similarities and differences between SLA and DLP

	Similarities	Differences			References
		Description	Advantages	Disadvantages	
**SLA**	Selective curing of bio-ink in a vat through light-activated polymerization Selective initiation of solidification in thin layers of liquid photopolymers using light sources Bio-ink and support structures required Preparation of photocrosslinked hydrogels commonly used in BTE	Laser photopolymerizes biomaterial within a vat in a raster-like contactless fashion	Average resolution (micrometer) Moderate printing speed Mild printing process Printing cost-effective	Long printing time for large size materials Volume shrinkage of the material Cause strong internal stresse Point-to-point linear aggregation	[21, 42–44]
**DLP**		planar build via projection of digital patterns into a stationary biomaterial vat	Great resolution (nanometer) Rapid printing speed Geometrically complex structures can be manufactured Visible light crosslinking can be utilized Scalable build volume	Mechanically weak at interfaces Opaque biomaterials limit light penetration and resolution Difficult to incorporate multimaterial print Only print small-sized materials	[21, 43–48]

### SLA

SLA was the first commercially available 3D printing technique and was invented by Charles Hull in 1986 [49]. This technology is considered to be the most mature and extensively used 3D printing technology in industry [45]. Lithography-based 3D bioprinting has unique advantages in BTE due to its printing speed, mild printing process and cost-effective features [21].

**Figure 1.**
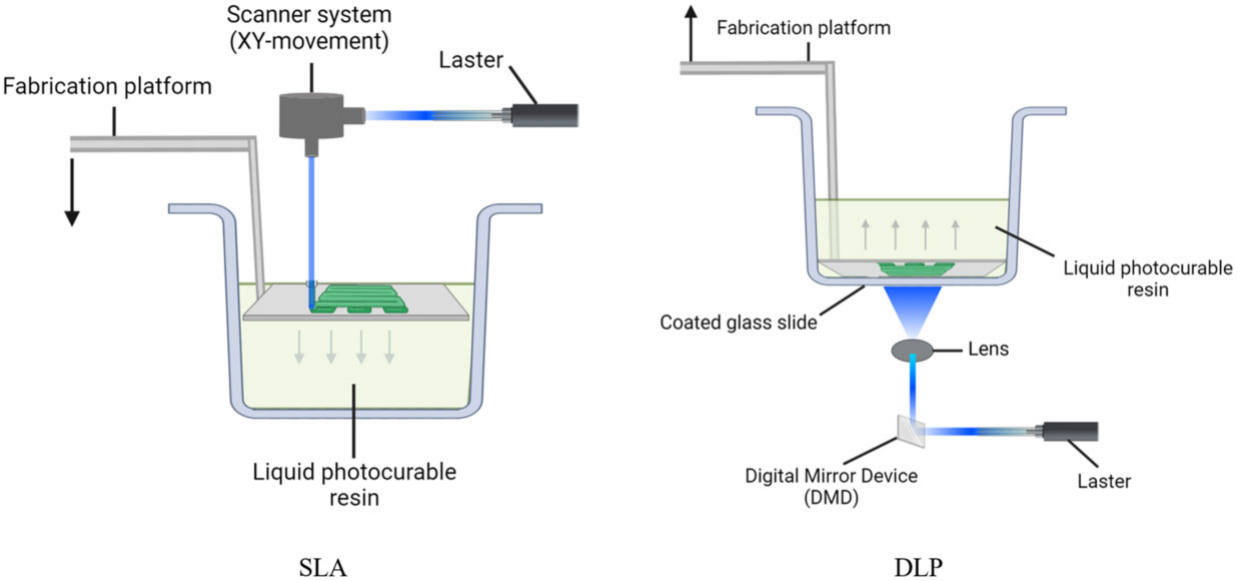
Schematic illustration of two 3D printing methods.

Specifically, the SLA setup comprises a reservoir holding photocrosslinkable liquid bioinks, a light source inducing hydrogel photopolymerization and crosslinking, a system allowing horizontal movement of the laser beam, and a platform controlling the material manufacture through vertical locomotion [21, 42]. The wavelength of the light source commonly used in SLA technology is a 355 nm laser beam, which is located above the liquid reservoir, and the exposure direction is from the top down. Bioink, a PCH, is solidified during the laser beam scanning process. Then, the platform descends into the bioinks, leading to the formation of the first layer attached to the platform. Afterward, the platform goes down a distance with one layer, and the uppermost layer is then covered with uncrosslinked bioink to print the next layer. These steps were repeated for printing until a solid PCHs-based scaffold was produced (Fig. 1) [21, 50].

Notably, the printing resolution relies on the size of the laser beam, and the pattern formation of each layer is controlled by its trajectory [45]. As a result, SLA may have a lower resolution than other photocuring technologies [51]. Due to the relatively small size of BTE scaffolds, the SLA printing rate should not be a concern. SLA is a technique based on vat polymerization that manufactures BTE scaffolds by selectively polymerizing photocrosslinkable materials [43]. This inevitably leads to a certain degree of volume shrinkage of the materials, and it could cause strong internal stresses, which may result in deformation or even fracture [44]. Based on full consideration of these problems, SLA can be widely used in BTE as it can develop cellular/bioactive component-laden porous scaffolds with PCHs.

### Digital light processing

DLP is another representative lithography-based 3D bioprinting technology characterized by a layer-by-layer-based printing pattern [46]. The core of DLP technology is the optical semiconductor, also called a digital micromirror device (DMD), invented by Dr Larry Hornback in 1977 and commercialized by Texas Instruments in 1996 [45].

The setup of DLP is similar to SLA, but the scanning galvanometer in DLP is substituted by a DMD. Currently, most light-based 3D printers use DLP technology controlled by a DMD. The DMD chip, providing superior image stability, fidelity and reliability, is a key component of DLP printing technology. This device comprises an array of millions of micromirrors, each representing one pixel in the digital image. Thus, microscale resolutions as small as 3–5 μm feature sizes can be achieved under the appropriate optics [52]. In brief, each micromirror can be individually rotated and created with an ‘on’ or ‘off’ state to control the reflection of the projected light. By regulating these different ‘on’ or ‘off’ states, different light patterns can be rapidly projected onto the liquid reservoir to enable selective solidification [21]. Therefore, instead of point-to-point linear polymerization of bioink, DLP, an improved version of SLA, cures one layer at a time [43, 44]. DLP printing can significantly reduce printing time compared to SLA (Fig. 1) [47].

Different from conventional extrusion and inkjet printing, DLP bioprinting technology is gentler on cells and bioactive agents because it does not require high temperatures or exerts shear stress [44]. Moreover, visible light instead of UV light can be used in DLP technology to crosslink bioinks, which is theoretically safer and less harmful to cells [48]. Cell distribution can also be well controlled in DLP-printed scaffolds [53]. Therefore, by increasing the DLP printing resolution or controlling the printing parameters, PCHs-based scaffolds can be generated with microscale, stratified structures or bioactivity. However, to ensure high-precision printing, DLP technology can only print small-sized materials due to the limited projection size [45]. It should be noted that impurities and inhibitors are critical factors in the hydrogel photocrosslinking process in DLP-based 3D bioprinting. In particular, oxygen impurities may diffuse into the materials over time, which can indirectly affect the printing performance of the material. In addition, the choice of photo-initiator is crucial, as it can determine the photocrosslinking efficiency, which in turn affects the printing time, power and resolution [54].

In general, SLA technology is more suitable for preparing large size materials due to the low print resolution caused by the reliance on laser beam. DLP, on the other hand, provides a finer resolution due to the addition of DMD, and the layer-by-layer printing makes the DLP printing process less damaging to cells with a faster printing speed. However, due to the limited projection scale, DLP is only capable of printing small-sized materials, and is more often used for BTE compared to SLA.

## Application in bone tissue engineering

### Natural polymers and mixtures based on natural polymers

Biocompatibility and biodegradability are the main advantages of natural polymers, which can effectively promote cell adhesion, migration, differentiation and tissue regeneration [30, 34, 55–57]. In addition, the insufficient mechanical properties of natural polymers can be slightly improved after photocrosslinking, making it possible to prepare BTE scaffolds with specific shapes through photolithography-based 3D bioprinting [58, 59]. Thus, the application of natural polymers, including gelatin methacrylate (GelMA) [60–62], methacrylated hyaluronic acid (HAMA) [63–65], silk methacrylate (SilMA) [24, 66–68], methacrylated chitosan (CSMA) [57, 69, 70], etc., has been widespread in BTE (Table 2).

**Table 2.** Characteristics and applications of different materials

Materials	Concentration (w/v)	Advantages	Disadvantages	Application	References
GelMA	5–10% (with cell) 15% (without well)	Satisfactory biocompatibility High viability of cells (83% and 73% at day 1 and 3) Acceptable printability Promotion of osteogenic differentiation	Poor compression strength (2–30 kPa) Short degradation time (32% and 52% at day 7 and 28 *in vivo*) High swelling rate	The formation of blood vessel Cranial defect in rats Drug and growth factor delivery	[58, 59, 71, 72]
HAMA	1.5% (with cell) 3% (without cell)	Satisfactory biocompatibility High viability of cells (73.6% and 64.4% at day 1 and 21) Promotion of osteogenic differentiation	Poor compression strength (1–10.6 kPa) Excessive degradation rate (80% at day 20 in PBS) High swelling rate Poor printability	Cranial defect in rats Cartilage defect of rabbit knee Growth factor delivery	[30, 63, 64]
SilMA	30% (with and without cells)	Satisfactory biocompatibility Satisfactory printability Acceptable compression strength (800–1000 kPa)	Low compressive modulus (10 kPa-200kPa) Short degradation time (50% at day 28 in PBS) High swelling rate (about 1000–2000%)	Meniscus regeneration Damaged trachea of rabbit Cartilage defect of rabbit knee Growth factor delivery (4D printing)	[24, 67, 68]
PEGDA	5–10% (with cell) 15% (without cell)	Nontoxic Anti-swelling Satisfactory printability High strength	Low viability of cells (printing with cells) Lack of cell adhesion sites Long degradation time Brittle	Cranial defect in rats Femoral condylar defect Cartilage defect of rabbit knee	[32, 73–75]
PPF	PPF: DEF = 3:1 (without cell)	Nontoxic Compression strength (about 15 mPa)	Long degradation time (more than 52 weeks) No mention of printing with cells into bioink	Cranial defect in rats Femoral condylar defect Bonding of fractured bones	[76–79]
F127DA	20% (without cell)	Nontoxic Anti-swelling (about 640%) Elastic and anti-fatigue (about 95%) Satisfactory printability	Low viability of cells (printing with cells) Lack of cell adhesion sites Long degradation time Low compressive modulus (80 kPa)	Drug delivery Bonding of fractured bones The formation of blood vessel Thyroid cartilage defects of rabbit	[80–83]

#### GelMA

Gelatin is a biodegradable polypeptide derived from the partial hydrolysis of collagen. It has been widely used in BTE due to its good biocompatibility, bioactivity and cell adhesiveness. Gelatin can possess the ability of photocrosslinking after being modified to GelMA by reaction with methacrylic anhydride (MA) [71]. GelMA is cytocompatible with similarity to the extracellular matrix. Therefore, it is suitable for 3D cell culture, as the cells encapsulated in GelMA have been shown to exhibit high cell viability [55]. However, different polymer concentrations (generally 10%) and different substitution rates (80%) may prevent cell proliferation in space. Therefore, GelMA with a degree of functionalization of 20–80% is used to generate stable hydrogels. Similarly, GelMA-based tissue engineering scaffolds with specific mechanical properties (from less than 10 kPa to more than 30 kPa) can be prepared by DLP printing technology with different polymer concentrations, substitution rates and initiator concentrations [58, 59].

**Figure 2.**
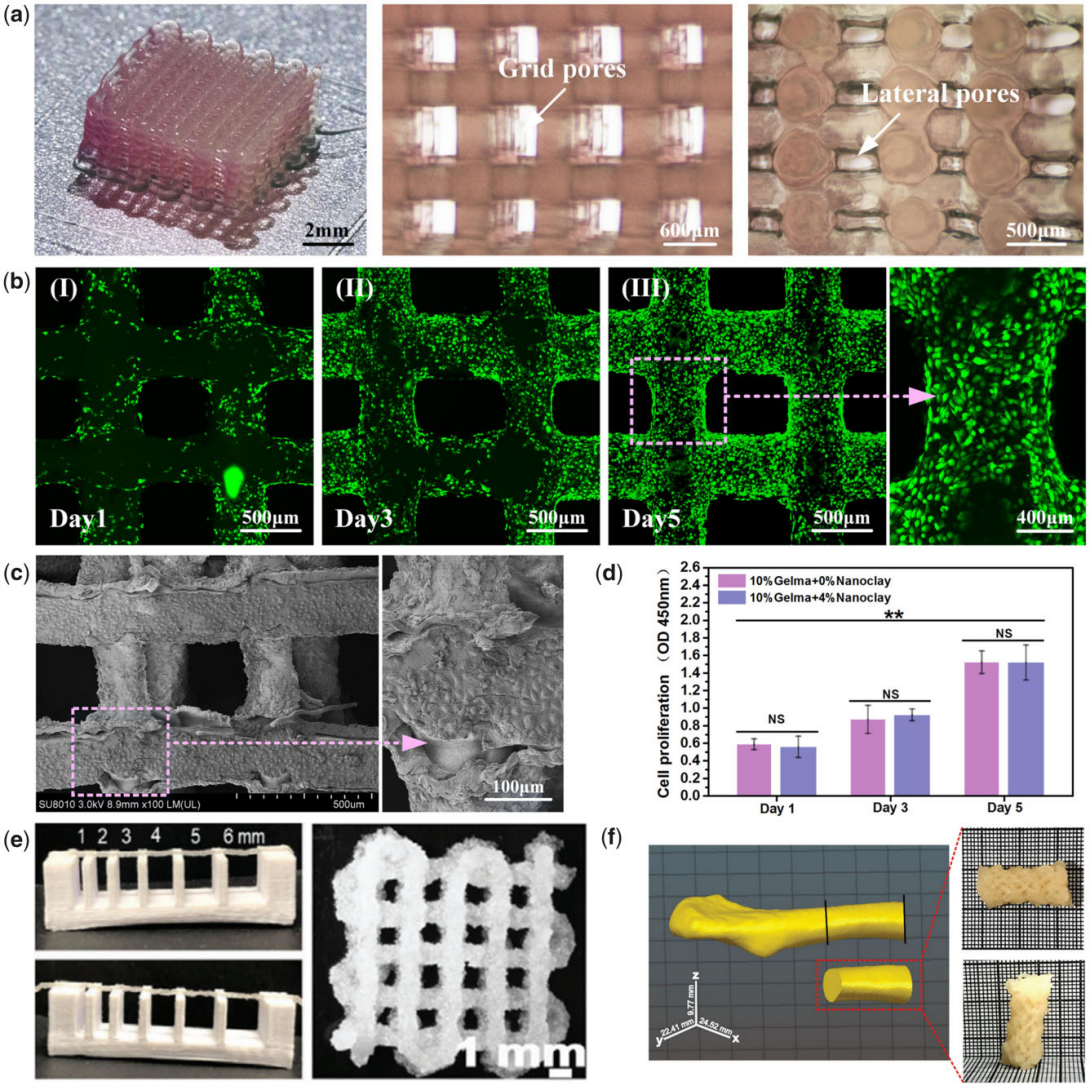
(**a**) Porosity of the GelMA/nanoclay scaffold. (**b**) Cell growth on scaffolds with 10%/4% GelMA/nanoclay on day 1 (I), day 3 (II) and day 5 (III). (c) Scanning electron microscopic image of scaffolds. (**d**) 3D image of cell proliferation on scaffolds. Reproduced from Gao *et al*. [61] with permission from copyright 2019 IOP publishing. (**e**) Filament collapse test of GelMA/BPs scaffold. (**f**) Physical integrity demonstrated by 3D model of patient-specific clavicle bone. Reproduced from Ratheesh *et al*. [62] with permission from Copyright 2020 Wiley.

Similar to the traditional hydrogel system, the main drawback of pure GelMA is that it has insufficient mechanical properties, which restricts its usage in BTE [71, 72, 84]. However, the addition of various nanomaterials and their physical or covalent combination with GelMA endow hydrogel scaffolds with better mechanical and biological properties [71]. Among them, mineral-based nanoparticles, such as hydroxyapatite and nanoclay, are still the most commonly used in BTE. Hydroxyapatite, as the most important inorganic component of bone, can increase the osteogenic capacity of GelMA scaffolds. Zuo *et al*. showed an increase in the compression modulus of hydrogels from ∼13 kPa to ∼23 kPa for pure GelMA to GelMA containing 2% (w/v) hydroxyapatite. In addition, after 7 days of coculture of different scaffolds and cells, the expression of most osteogenic genes in the scaffolds containing hydroxyapatite increased [71]. Gao *et al*. designed the printing strategy of nanoclay/GelMA composite hydrogels, divided the printing process into three stages, extrusion, deposition and fusion, and gave specific printing parameters as they systematically studied the influence of manufacturing process parameters on the support forming process (Fig. 2a–d) [61]. Moreover, Greeshma *et al*. prepared a GelMA-based bioink with autologous bone particles (BPs) and determined appropriate printing parameters after a series of tests, including rheological analysis and mechanical performance evaluation. Finally, it was found that GelMA/BP composite scaffolds based on 3D printing could effectively promote bone regeneration by observing the proliferation, migration and osteogenic differentiation ability of cells in the scaffolds (Fig. 2e and f) [62].

#### HAMA

**Figure 3.**
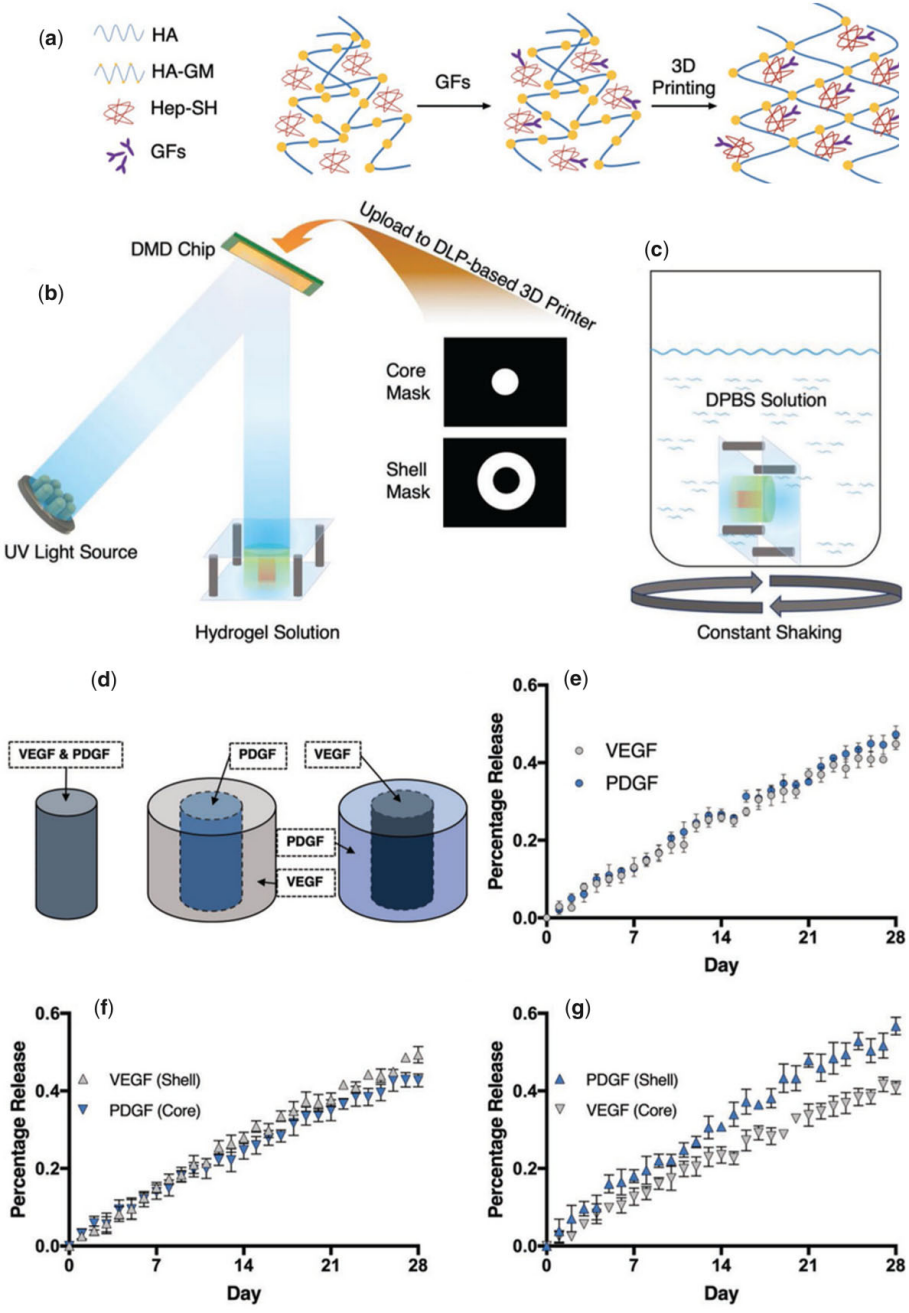
Controlled growth factor release in 3D-printed hydrogels: (**a**) Schematic of synthesis and polymerization; (**b**) process of DLP-based 3D printing; (**c**) the hydrogels were immersed into DPBS solution to study GF release kinetics; (**d**) core–shell structure; (**e**–**g**) the release of VEGF and PDGF. Reproduced from Wang *et al*. [63] with permission from Copyright 2020 Wiley.

Hyaluronic acid (HA), similar to gelatin, is abundant in the extracellular matrix of bone and provides mechanical support. HA can be modified by methacrylate groups to obtain HAMA, endowed with photocrosslinking properties, improving printability and rigidity while maintaining fine biocompatibility [56]. Poldevaart *et al*. demonstrated that mesenchymal stem cells derived from human bone marrow could achieve 21 days of survival and osteogenic differentiation in 3D-printed HAMA scaffolds. Although the effectiveness of scaffolds in animals has not yet been proven, it is enough that 3D-printed HAMA scaffolds could be a feasible scheme in BTE [30]. In addition, 3D-printed HAMA scaffolds can also be used as a carrier for growth factors to slowly release factors. Wang *et al*. proved that the compound bioink for 3D printing comprised of HAMA, thiolated heparin (Hep-SH) and growth factors could control the release rate of growth factors in hydrogels by the different geometry and spatial ordering (Fig. 3) [63].

Despite its good biocompatibility, HAMA is often used in combination with other materials (i.e. polymers) in BTE on account of the deficiency of mechanical properties required for bioprinting [64]. Cristina *et al*. mixed HAMA, alginate and PLA to obtain a 3D printing hydrogel scaffold, which is expected to promote bone tissue regeneration through endochondral osteogenesis, with good mechanical properties, including printability, gelling ability, stiffness and good degradability [65].

#### SilMA

**Figure 4.**
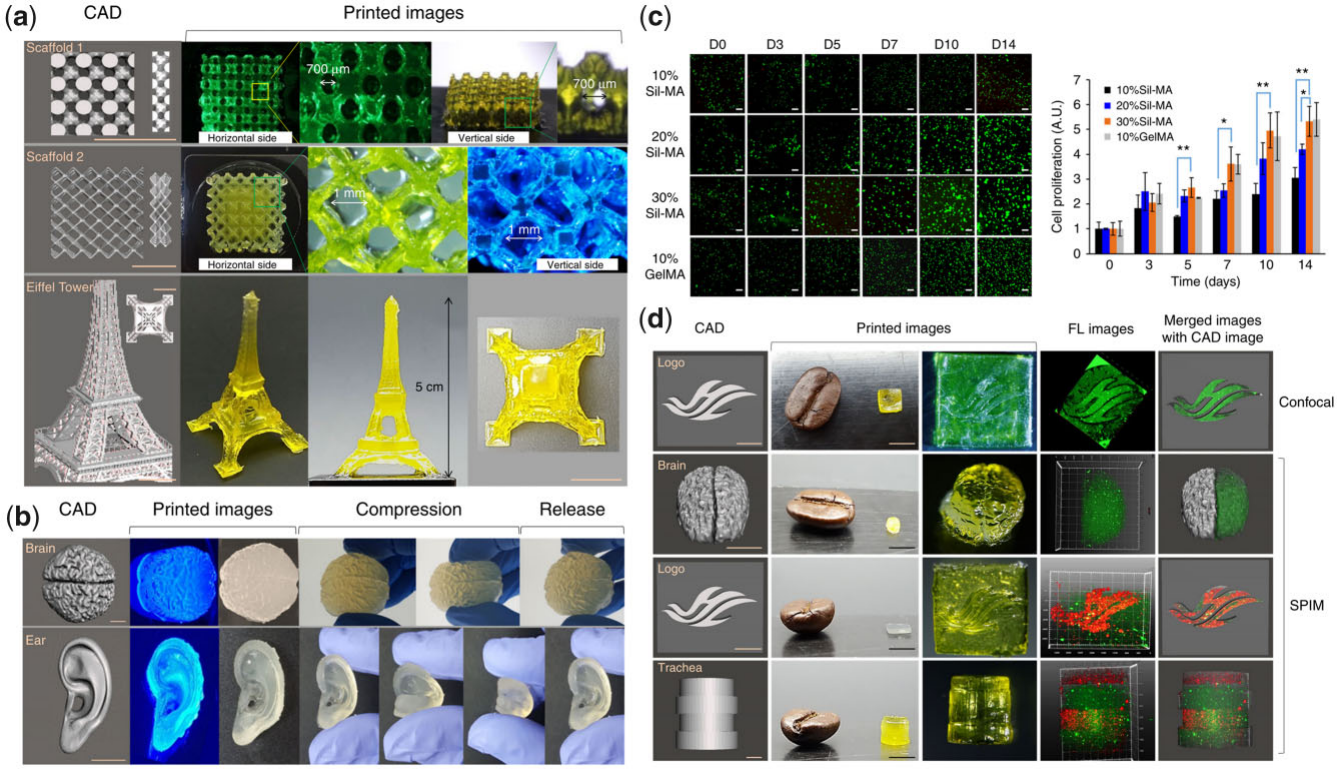
Printing performance of SilMA and its biocompatibility: (**a**) CAD and printed images of porous scaffolds and Eiffel Tower. (**b**) CAD, printed images and compressed performance of brain and ear. Scale bar indicates 1 cm. (**c**) Live and dead stain and CCK-8 assay to evaluate the proliferation of NIH/3T3 for 14 days. (**d**) Cell distribution inside the Sil-MA. Scale bar indicates 1 mm on CAD images and 5 mm. Reproduced from Kim *et al*. [68] with permission from Copyright 2018 Springer Nature.

SF, one of the strongest fibrous proteins in nature with excellent biocompatibility and biodegradability, can be chemically modified through methacrylation to prepare SilMA for the application of DLP printing technology [67]. Kim *et al*. [68] demonstrated a technique to produce an effective SilMA-based bioink with not only satisfactory printable properties but also good cytocompatibility by glycidyl methacrylate (GMA). The 30% SilMA is used to prepare different complex models (e.g. tissue-engineered scaffolds, brains, ears) (Fig. 4a) by DLP printing technology with better mechanical properties than ordinary hydrogels, as the brain and ear could return to their original shape after distorted by pressure (Fig. 4b). In addition, NIH/3T3 fibroblasts were added to bioink for DLP printing and then cultured *in vitro*, with the majority of cells remaining viable at 14 days, regardless of SilMA concentration (Fig. 4c and d). These properties of the stent are essential for its application in BTE.

Not only can SilMA be used alone for DLP printing, but it can also be combined with other materials to prepare bioink. Bandyopadhyay *et al*. [24] mixed different concentrations of SilMA with PEGDA with the addition of chondrocytes to construct 3D bioprinted osteochondral scaffolds, which possessed a porous structure inside the scaffold with biodegradable property. An increased secretion of cartilage-associated proteins could be observed after culturing chondrocytes laden on the scaffold. These properties mentioned above suggest the ability of SilMA-PEGDA to be applied to cartilage regeneration and the potential for application to BTE, as endochondral ossification can also be one of the ways for bone regeneration.

SilMA with good cytocompatibility and printing performance has been used in cartilage tissue engineering in most studies so far, however, its advantages in BTE also need to be explored in terms of its properties.

### Synthetic polymers and mixtures based on synthetic polymers

Although natural polymers have good biodegradability and biocompatibility, they are mainly used as additives or composites in BTE in that their mechanical properties do not reach the normal level of cancellous bone (>100 MPa) [64, 85, 86]. Therefore, in BTE, synthetic polymers with higher compression strength are often used as the main material of scaffolds together with natural polymer materials with excellent bone induction properties to meet the needs of structural and functional bionics [87]. At present, the synthetic polymers suitable for 3D printing mainly include poly(ethylene glycol) diacrylate (PEGDA) [73–75, 88], PPF [89–91], pluronic F127 diacrylate (F127DA) [80–83], etc. (Table 2).

#### PEGDA

**Figure 5.**
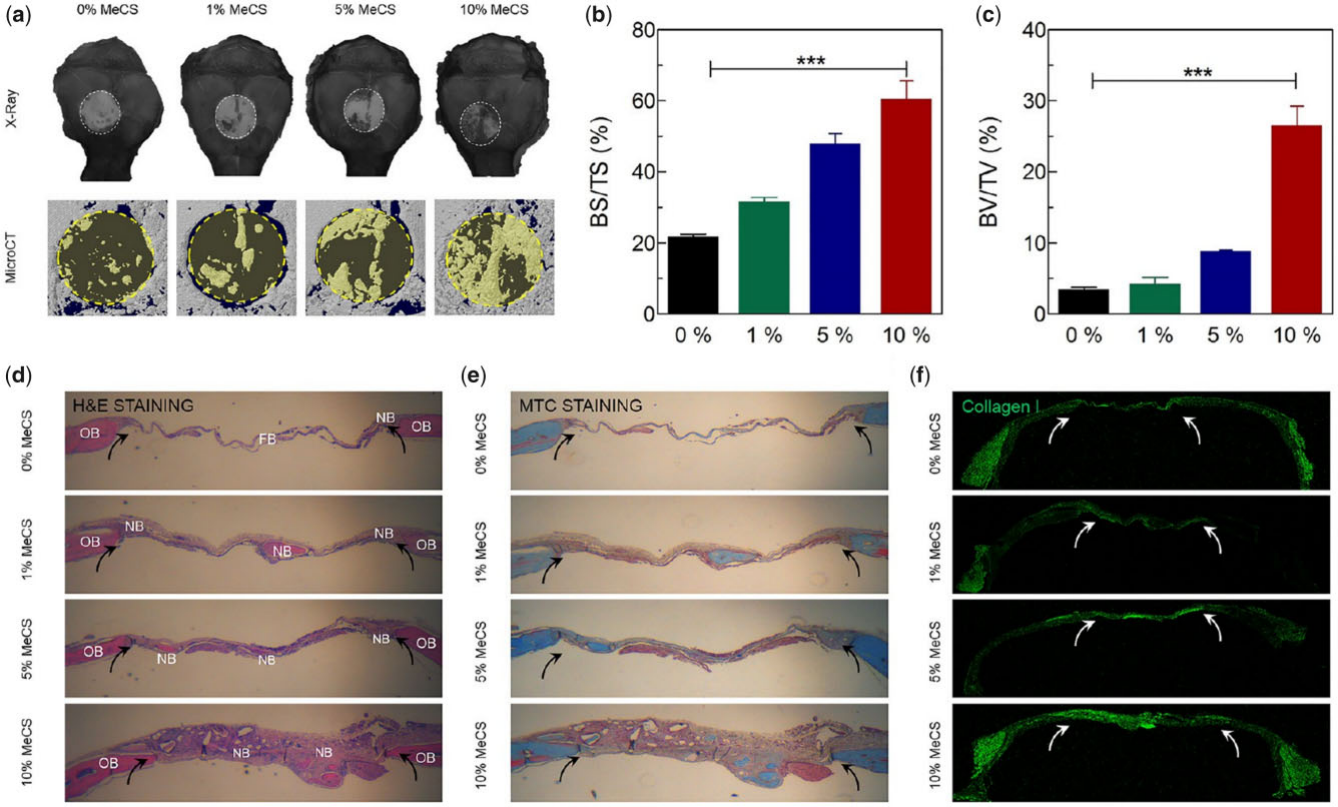
*In vivo* bone regeneration. The hydrogels ladened with cells were transplanted into a 4 mm-diameter defect of mouse skull. (**a**–**c**) X-ray and micro-CT analysis of bone repair *in vivo*. (**d**–**f**) Histological analysis with H&E, MTC and collagen I staining. Reproduced from Kim *et al*. [78] with permission from Copyright 2017 American Chemical Society.

PEG, approved by the Food and Drug Administration (FDA) for various biomedical applications, exhibits high biocompatibility and almost no immunogenicity [92, 93]. PEGDA with light crosslinking properties is formed after the modification of PEG, which has low viscosity and high solubility, making it an ideal biomaterial for 3D bioprinting [75, 88]. PEGDA scaffolds with specific physical and mechanical properties can be prepared by adjusting the molecular weight or concentration of PEGDA, and the degradation rate can be controlled by changing the degree of polymerization [32]. Kim *et al*. manufactured PEGDA/chondroitin sulfate 3D printing hydrogel scaffolds that can induce osteogenic differentiation of bone marrow-derived mesenchymal stem cells *in vitro*. PEGDA provided good printability for scaffolds, while chondroitin sulfate combined with calcium, phosphorus and other charged ions to form a microenvironment conducive to osteogenesis by the negative charge on its sulfate group. The composite hydrogel scaffold notably promoted bone regeneration after implantation into a critical size defect of the rat skull (Fig. 5) [74].

In addition, the combination of minerals and PEGDA is also a good choice to improve mechanical and biological properties. Gaharwar *et al*. suspended hydroxyapatite nanoparticles into bioinks to add more hydroxyapatite into hydrogel scaffolds while maintaining 3D printing capability. The results showed that the addition of less than 15% hydroxyapatite nanoparticles did not significantly change the printing capability of the hydrogel scaffold but improved the mechanical properties, including a 3-fold increase in tensile modulus, an 8-fold increase in fracture strength and a 10-fold increase in toughness. The swelling degree also decreased with increasing concentrations of hydroxyapatite nanoparticles, although the shape remained unchanged [73].

Although there are many advantages of PEGDA, it is generally inelastic and brittle, which makes it more likely to be used in combination with other materials rather than alone for BTE [94].

#### PPF

As a biodegradable polymer, PPF is degraded by hydrolysis of its ester bonds, with fumaric acid and propylene glycol as nontoxic degradation products, which has been extensively studied in the past decades due to their promising biocompatibility and controlled mechanical properties [76, 77, 95]. PPF can be dissolved in solvent (DEF) and crosslinked in the presence of photoinitiator (BAPO). PPF was used for BTE in 1996 [78] and until 2003 when Cooke *et al*. prepared tissue engineering scaffolds to repair defects by SLA printing technique, PPF has been widely used in BTE [79, 89].

**Figure 6.**
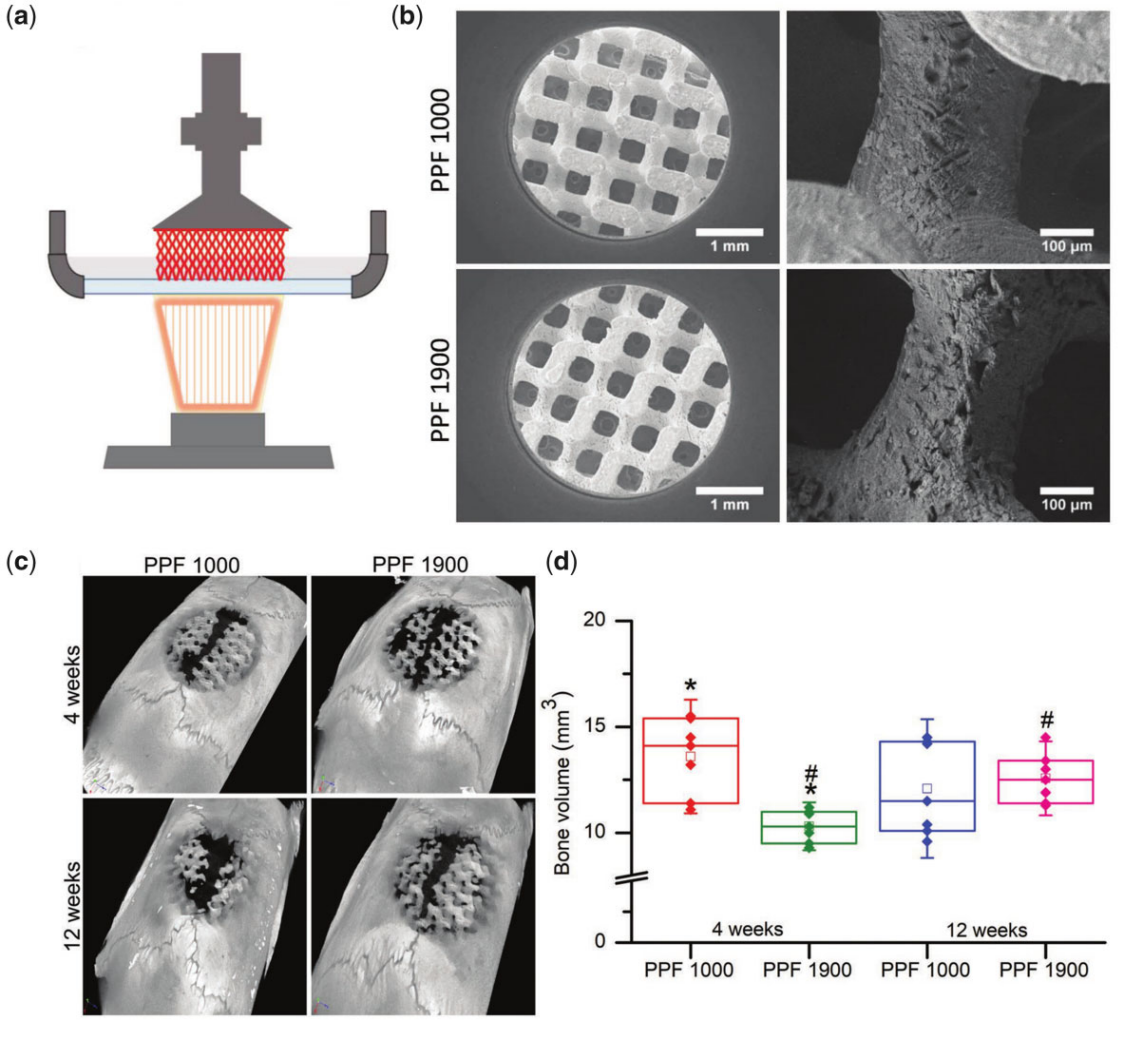
3D printed scaffolds and their regenerative bone volume: (**a**) preparation of complex structures by DLP technology; (**b**) SEM images (25× and 200×) of scaffolds; (**c**) 3D images of the defect area; (**d**) bone volume data with box plots for all time points and groups. Reproduced from Nettleton *et al*. [83] with permission from Copyright 2019 Wiley.

One of the concerns about the use of polymers in BTE is the degradation time. In order to control the degradation rate of the scaffold, Nettleton *et al*. [91] prepared PPF (1000 and 1900 Da) with different relative molecular masses as bioinks, printed them by DLP technique and implanted them into rat cranial defects to study their degradation properties and bone regeneration ability. PPF with 1000 Da formulation at 4 W degraded more faster than PPF with 1900 Da formulation, resulting in more bone tissue into the defect site; at 12 W, the amount of regenerated bone with low molecular mass PPF was similar to that with high molecular mass PPF (Fig. 6). Therefore, it is crucial to have an appropriate degradation rate for the repair of bone defects.

Not only that, PPF can also be used as a carrier of cells or factors [90, 96, 97]. It is possible for the cells to adhere, proliferate and differentiate on the scaffold; furthermore, the PPF-based scaffold enables the slow release of growth factors (TGF-β, BMP-2). With two important characteristics of biocompatibility and biodegradability for tissue engineering, PPF-based scaffolds can be prepared by SLA or DLP printing and has been widely used in BTE.

#### F127DA

With both temperature and photosensitive properties, F127DA can achieve reversible conversion between liquid and solid phases from room temperature to 37°C. It can also form a stable hydrogel at room temperature by crosslinking under the stimulation of photoinitiators. F127DA is widely used in tissue engineering due to its low swelling rate, high strength, fatigue resistance and appropriate elastic modulus [83]. Shen *et al*. [80] prepared microfluidic chips based on the anti-fatigue and anti-swelling properties of F127DA, and the inner part of them was inoculated with HUVEC to promote endothelial functional expression by perfusion culture. In addition, they further prepared vascular spheroids, which are expected to be applied in BTE to promote vascularized regeneration [82].

Despite its several advantages, F127DA alone is not conducive to cell adhesion due to the lack of cell adhesion sites. Therefore, F127DA-based composites are more favorable for adhesion to cells. Ren *et al*. [81] added 1.5% HAMA to F127DA bioink, which increased the bioactivity of the scaffold while its mechanical properties were retaining, and good therapeutic effects were observed after implantation into rabbit thyroid cartilage defect sites. In addition, by combining sodium alginate with F127DA, Wang *et al*. [98] prepared a shape memory hydrogel with good biocompatibility through 3D printing, which has good prospects for drug delivery.

At present, F127DA is rarely used in BTE, which may be related to its poor cell adhesion. Although some scholars have solved this problem by RGD peptide modification [99], more researches are needed to make F127DA more useful in bone regeneration.

There are also some polymers, such as PCL, PLA, etc., which are more often using FDM printing technology rather than DLP or SLA for the preparation of the scaffolds. In addition, some ceramics such as HA, TCP powders can also be printed using DLP technology, but it requires the use of a photosensitive resin because it does not have its own photocrosslinking properties. The powder is dispersed in a photopolymer resin and then printed, and the resulting mold is removed of organics and sintered (600°C) to obtain the scaffold [100].

## Summary and prospects

PCHs, which are easy to prepare, are attractive because of the inherent superiority of light as a stimulus in BTE. Light is noninvasive with limited byproducts, which allows remote manipulation of materials without additional reagents [54]. Hydrogels with photoresponsive functionalities enable the most precise spatiotemporal control over the reaction of functional groups. In terms of the material itself, the density of photocrosslinking can be adjusted by the material concentration or the substitution rate of photoreactive groups. In addition, regarding the operation method, the degree of photocrosslinking can be regulated by the irradiation parameters, such as light intensity and irradiation time [101]. Thus, PCHs are more likely to simultaneously possess biocompatibility, biodegradability, ossification and mechanical properties, making them outstanding materials in BTE. Bone, as a complex natural biomineralization system, possesses a complex composition, special structure and diverse functions. Therefore, the preparation of BTE scaffolds that can successfully promote bone regeneration must be based on an in-depth understanding of natural bone.

In terms of the complex composition, natural bone consisting of the soft matrix (collagen-I) and rigid matrix (hydroxyapatite) make the selection of materials crucial for BTE [102]. Natural polymer hydrogels with good biocompatibility and degradability lack sufficient mechanical properties, while synthetic polymer hydrogels possess satisfactory mechanical strength similar to natural bone with deficient osteogenic ability. Thus, different materials have different application scenarios (different types of bone defects). In inclusive bone defects, the scaffold needs to possess osteoconductive capacity and controlled degradability, which is not a high requirement for the compression strength of the scaffold, making natural polymers perhaps a good choice. In contrast, segmental bone defects require the scaffold with certain mechanical properties during the first and middle stages of bone regeneration, which is why synthetic polymers or composites are necessary to meet the demand. Although a variety of PCHs already have been developed, there is still a need to develop new materials or modify existing materials to meet the various requirements of BTE in terms of mechanical properties, biocompatibility, biodegradability, etc.

In terms of the special structure, the complex hierarchical structure of natural bone tissue, i.e., macrostructure and microstructure. On the macroscopic level (micromillimeter scale), bone tissue can be divided into two parts according to its structure. Part of the structure containing trabecular bone is cancellous bone, accounting for approximately 20% of the entire skeletal system with a porosity of approximately 50–90%, which is loose in structure like a spongy mesh in which the center is filled with bone marrow, nerves and blood vessels. The other part containing multiple layers of closely arranged bone plates is cortical bone or compact bone, accounting for approximately 80% with only 10% porosity, which is dense in structure like a thick-walled cylinder with the distribution on the surface of the bone [103, 104]. On the microscopic level (nanoscale), bone is mainly composed of type I collagen and nanohydroxyapatite, which are regularly arranged in a quarter-staggered array with a period of approximately 67 nm [105]. Photocrosslinked hydrogel-based scaffolds prepared by photolithography-based 3D bioprinting technology can possess a biomimetic macrostructure of natural bone with desirable pore size and porosity. Thus, it can be produced in a favorable environment for bone regeneration, promoting the adhesion, proliferation, differentiation of cells as well as the formation of new bone. However, the bionic macrostructure of bone tissue may be easily accomplished by photolithography-based 3D bioprinting technology, but for the microstructure, the realization of bionics requires a more sophisticated manufacturing process. Although it has been suggested that DLP can print with the precision of nanometer, yet, no one has so far printed the microstructure of bone tissue and applied it in BTE. Therefore, simultaneously satisfying the macroscopic and microscopic principles of scaffolds may be the focus of future research on bone tissue engineering.

In terms of diverse functions, natural bone not only has its own compositions and structures but also contains a variety of cells, cytokines and active proteins, which play vital roles in regulating inflammation, promoting blood vessel and nerve remodeling, etc. Thus, these functional factors must be considered during the preparation of BTE as they play an important role in bone regeneration. Moreover, as one of the biological 3D printing methods, photolithography-based 3D printing can add a variety of cells and factors to PCHs-based bioinks while maintaining the activities of cells and factors during and after the preparation of the scaffold. However, the cells added to bioink at this stage are all 2D cultured cells *in vitro*, which cannot mimic the real *in vivo* environment, although they can also play a role in promoting bone regeneration. Based on this, cell spheroids and organoids have been rapidly developed. The cells aggregate to form micro-tissues based on extracellular matrix interactions in the absence of a fixed medium, thus cell spheroids or organoids are physiologically more similar to *in vivo* [106, 107]. Due to the 3D spatial structure, cell spheres or organoids possess superior biological properties than 2D-cultured cells, such as better cellular activity and proliferation, more stable morphology and polarization and excellent metabolic functions [108]. Siddharth *et al*. [109] combined cell spheroids with tissue engineering scaffolds to effectively promote bone tissue regeneration. Gabriella *et al*. [110] successfully cultured organoid of bone tissue *in vitro* and implanted them into murine tibial defects, the regenerated bone showed morphological characteristics similar to those of natural tibial bone. Both cellular spheroids and organoids showed good functional conditions, and we believe that it is likely to make outstanding contributions in the field of BTE, if photolithography could be effectively combined with cell spheroids or organoids and endow them with designable structural features. For instance, an ideal scaffold can be printed by lithographic 3D printing with cells mixed into bioink, while the cells can form spheroids even organoids on the scaffold, thus effectively promoting the regeneration of bone tissue.

In summary, BTE scaffolds prepared by PCHs based on photolithography 3D bioprinting technology can imitate the composition, macrostructure and functional characteristics of natural bone tissue well, achieving promising results for applications in BTE. However, it should not be ignored that the choice of material should be based on the specific type of bone defect, and the bionics of the microstructure need to be further explored for better application in BTE. In addition, it may be one of the trends to prepare scaffolds which can form cell spheres or organoids *in situ* by lithography using appropriate PCHs.

## Availability of data and material

The datasets used and analyzed during the current study are available from the corresponding author on reasonable request.
